# Novel surgical approach without bowel resection for multiple gastrointestinal lipomatosis: A case report

**DOI:** 10.1016/j.ijscr.2019.05.005

**Published:** 2019-05-10

**Authors:** Yasunori Yoshimoto, Tomoharu Yoshida, Takahisa Fujikawa, Yasuyuki Shirai, Tsunenori Yamamoto

**Affiliations:** a Tobata Kyoritsu Hospital, Department of Surgery, 2-5-1, Sawami, Tobata-ku, Kitakyushu, Fukuoka, 804-0093, Japan; b Kokura Memorial Hospital, Department of Surgery, Japan; c Kokura Memorial Hospital, Department of Gastroenterology, Japan

**Keywords:** Multiple gastrointestinal lipomatosis, Intussusception, Local excision, Endoscopic submucosal dissection (ESD)

## Abstract

**Introduction:**

Asymptomatic lipoma only requires observation, whereas symptomatic lipoma requires treatment such as endoscopic or surgical resection. However, in case of multiple lipomas, with evident diffusion and malignancy, resection procedure and range cannot be determined.

We experienced GI lipomatosis (multiple lipomas) diffusely existing from the duodenum to the small intestine and involved recurrent intussusception.

**Presentation of case:**

47 year-old female was a history of open bowel resection for intestinal obstruction caused by intussusceptions of multiple small intestinal lipoma 11 years ago. EGD showed duodenal lipoma, and CT showed diffuse multiple lipomas from the proximal jejunum to the distal ileum. Another CT also showed intussusception of small intestine, but no signs of intestinal obstruction. Surgical procedures performed included diagnostic laparoscopy. All intestinal lipomas were resected with local excision, and duodenal lipoma was resected with ESD without any bowel resection.

**Discussion:**

Multiple local excision  ESD for multiple GI lipomatosis have not been reported. The most problematic thing is that if extensive resection is performed to cut off all multiple lipoma, short bowel syndrome may occur. Determining the range to be cut remains unclear.

**Conclusions:**

Multiple local excision ± ESD seemed to be one of the methods in resecting multiple GI lipomatosis. In the future, cases and indications of surgery and resection method for GI lipoma should be accumulated and considered, respectively.

## Highlights

• We experienced GI lipomatosis existing from the duodenum to the small intestine.
• In case of multiple lipomas, resection procedure and range cannot be determined.
• Intestinal lipomas were resected with local excision without any bowel resection.
• And duodenal lipoma was resected with ESD.
• Local excision + ESD seemed to be one of the methods in resecting lipomatosis.

## 1 Introduction

Gastrointestinal (GI) lipoma is a rare disease with an incidence at autopsy ranging from 0.04% to 4.5% [1], and duodenal lipoma is also very rare; only 26 duodenal lipomas (0.023%) were found in 115251 routine autopsies [2]. The aetiology of diffuse infiltrating GI lipoma remains unknown; however, metabolic abnormalities, alcohol abuse, polyneuropathy, and malignancies have been described as possible causes [3]. GI lipoma is usually a single and slow growing benign tumour. Invasive management is not advised unless complications arise such as intussusception, obstruction, bleeding, or perforation leading to peritonitis. Intussusception is the most common idiopathic cause of bowel obstruction in certain paediatric population (6-36months) but vary rarely occurs in adults; 90% of cases have an organic cause, 60% due to neoplasm (60% malignant and 40% benign) [4]. Asymptomatic lipoma only requires monitoring, whereas symptomatic lipoma requires treatment such as endoscopic or surgical resection. However, in case of multiple lipomas, combination of diffuse and malignancy, both resection procedure and range should be performed.

We experienced GI lipomatosis (multiple lipomas) existing diffusely from the duodenum to the small intestine and involved recurrent intussusception. There was no finding suggestive of malignancy at the time of palpation. All intestinal lipomas were resected with local excision, and duodenal lipoma was resected with ESD without any bowel resection, as a considerable amount of small intestinal resection complicates postoperative short bowel syndrome.

No previous studies have reported the use of multiple local excision + ESD for the treatment of multiple lipomatosis; therefore, this case is reported along with a discussion of the relevant literature. This case report has been reported in line with the SCARE criteria [5].

## 2 Presentation of case

**Fig. 1:**
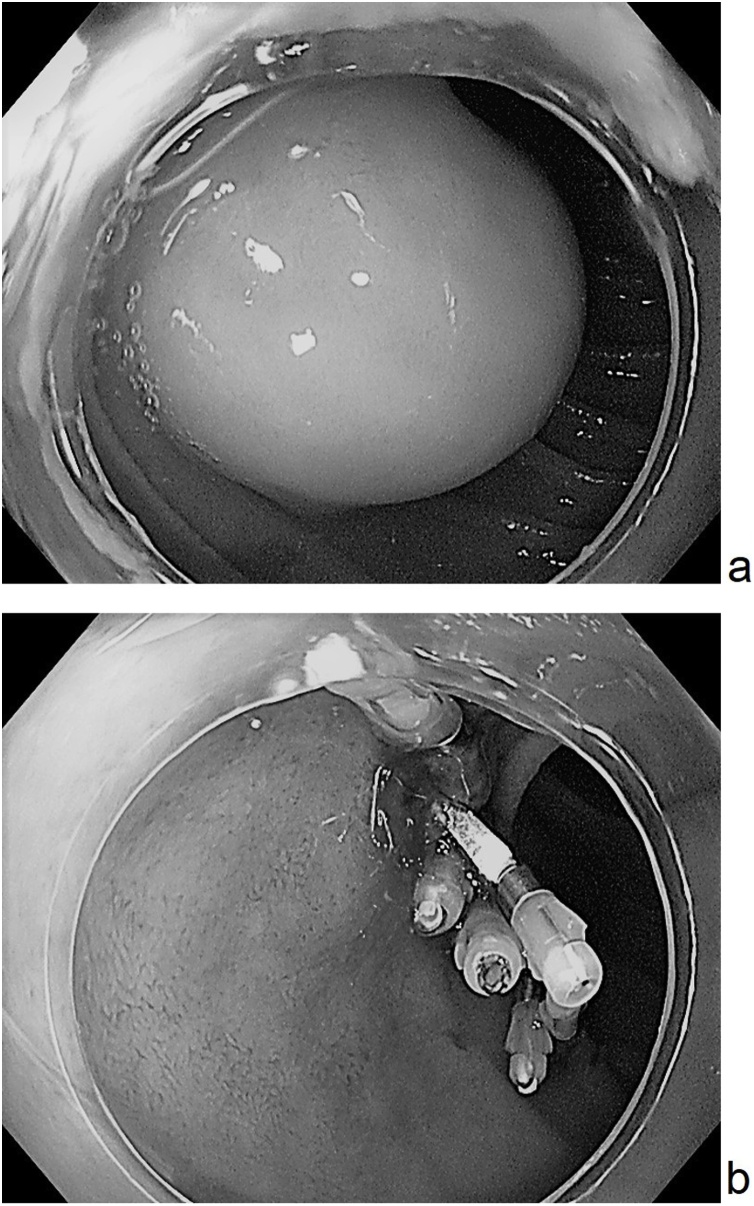
(a) GD showed the 20-mm duodenal lipoma, (b) Duodenal lipoma was resected by ESD.

**Fig. 2:**
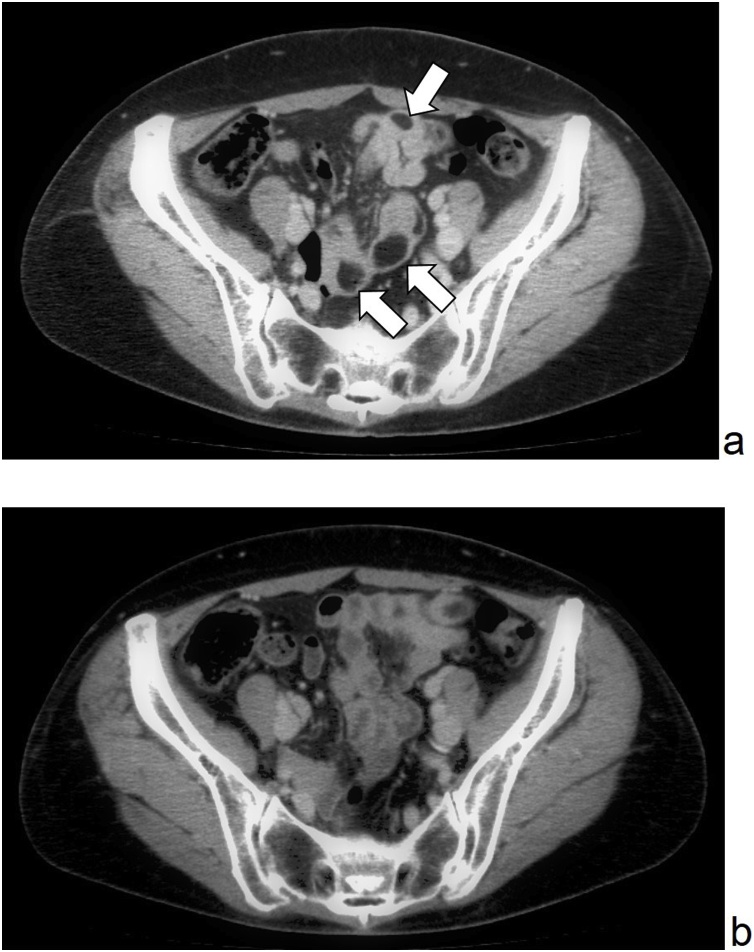
(a) Diffuse multiple lipoma (allow) was found from the proximal jejunum to distal ileum in pre-operative CT.(b) No obvious recurrence or increase of lipoma have been observed in post-operative follow-up CT.

**Fig. 3:**
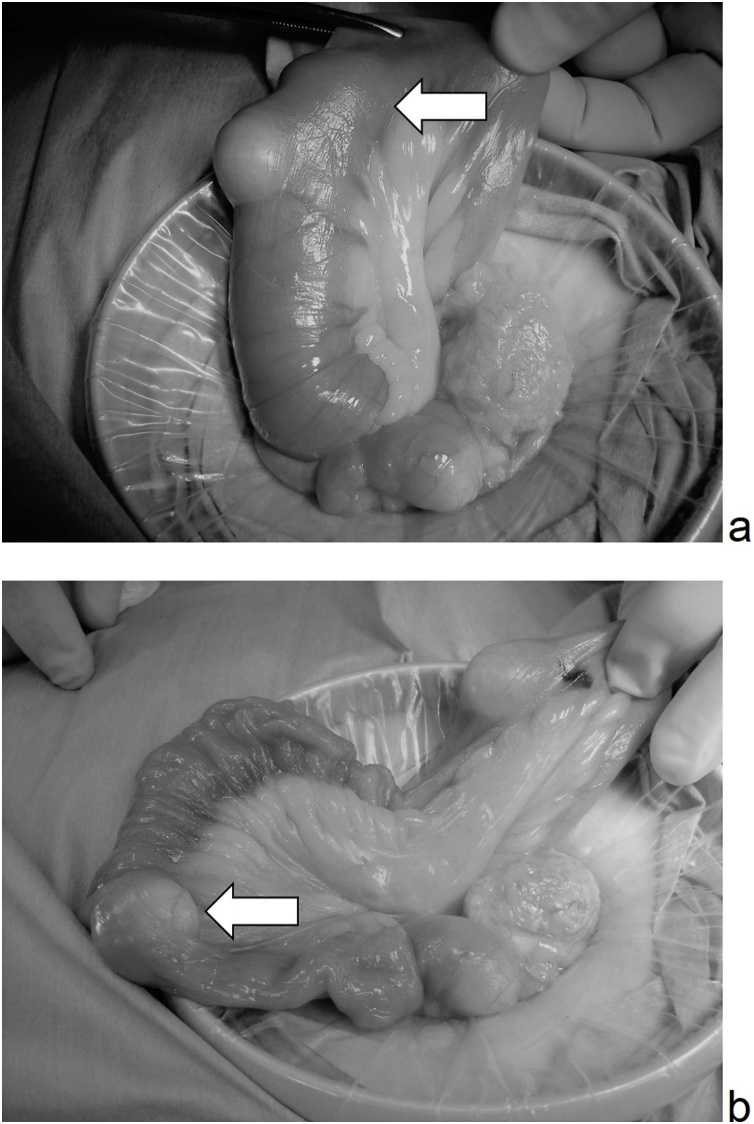
Operation finding showed (a) the small intestine on the side of the mouth was overlaid with lipoma at the tip (allow), and (b) the intussusception was easily reduced by manual Hutchinson procedure.

**Fig. 4:**
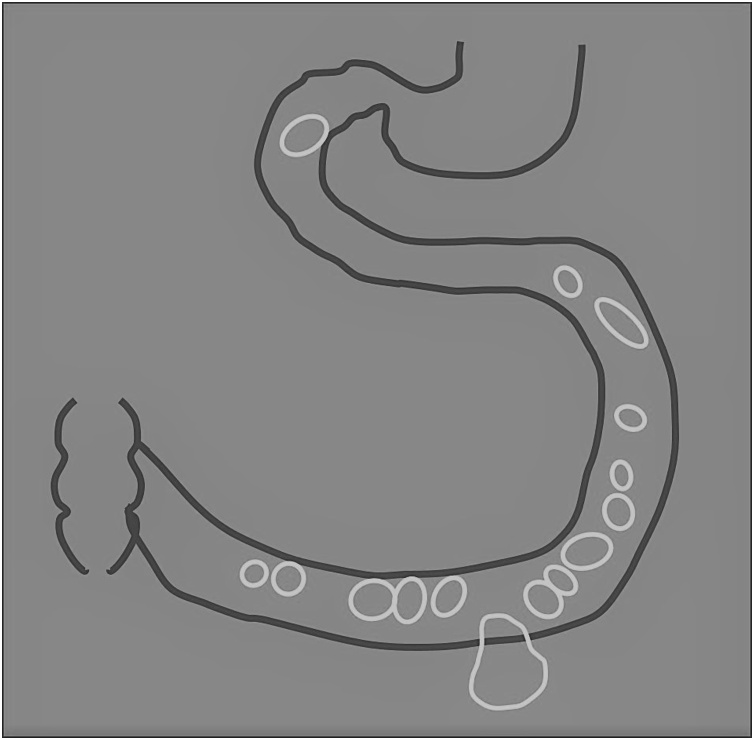
Schema of location of multiple GI lipomas.

**Fig. 5:**
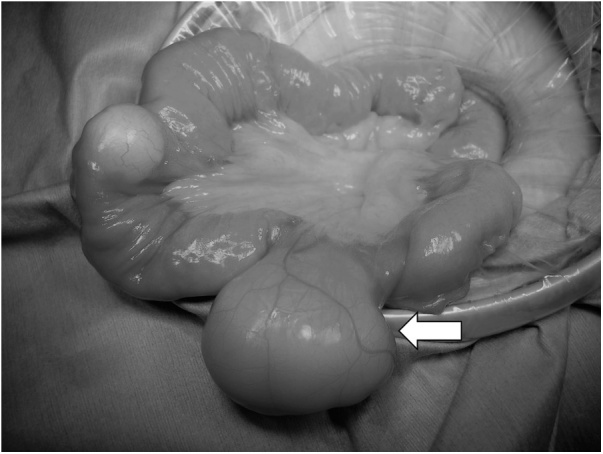
Operation finding showed the maximum size was 43 mm and exceeded the serosa.

Herein, we present a case of a healthy 47-year-old woman. In 2005, she underwent open bowel resection for intestinal obstruction caused by intussusceptions of multiple small intestinal lipoma. No other major complications were observed. Recently, she complained of abdominal pain, and after careful examination, EGD showed the 20-mm duodenal lipoma (Fig. 1a), and diffuse multiple lipoma (Fig. 2a) was found from the proximal jejunum to distal ileum in CT. Another CT also showed small intestine intussusception, but no signs of intestinal obstruction. The size exceeded the maximum 40 mm, identified as intussusceptions at one portion of the small intestine. Therefore, it was judged as conservative treatment limit and surgical treatment was selected. The surgical procedures performed included a diagnostic laparoscopy converted to open laparotomy. Initially, trocars were inserted to the umbilicus through an open method and observed inside the abdominal cavity. As a result, no obvious adhesion was detected, and the small intestine was good in mobility, and continued surgery from small laparotomy. There was mild adhesion in the vicinity of the last anastomotic site of 120 cm from the terminal ileum, and the small intestine on the side of the mouth was overlaid with lipoma at the tip (Fig. 3a). The intussusception was easily reduced by manual Hutchinson procedure (Fig. 3b). Lipoma diffusely exists from the 30 cm of the anal side in the Treitz ligament to 90 cm of the oral side from the terminal ileum, and approximately 15 lipomas considered as ≥10 mm were detected (Fig. 4). The maximum size was 43 mm and exceeded the serosa (Fig. 5). No tumor was found to suggest obvious malignancy on palpation. Basically, a small incision right above the tumor was made, the tumor was removed, and the mucosal and serous membrane/muscle layer were sutured. A suture that was very close could open two tumors from one incision. After completion the resection of the small intestine, it was immediately clamped just after Treize ligament, and duodenal lipoma was resected by ESD. The bleeding was stopped by a clip (Fig. 1b). The postoperative course was uneventful, and she started taking meals from postoperative day (POD) 2, and she were discharged on POD5. Pathological results showed that mature adipose tissue increased in the area considered to be directly under the muscular layer of mucosa. A tumour found was thought to be in or behind the proper muscle layer. To date, no obvious abdominal symptoms due to recurrence or increase of lipoma have been observed clinically or during follow-up (Fig. 2b).

## 3 Discussion

With the recent advances in endoscopy and modern imaging technologies such as CT scan and MRI, more cases of lipoma are being diagnosed and treated. Current imaging modalities such as CT and MRI can provide an accurate diagnosis [6]. GI lipoma can be easily diagnosed in recent years, which is relatively easy to distinguish between benign and malignant tumours. Although most lipomas are benign, removal of any intraluminal lipoma should be performed only because of possible future symptoms and malignancy.

Symptoms observed in the GI lipoma become an indication for resection, and the method and range of resection remain controversial. The localized, small, solitary lipoma can be easily and safely removed using an endoscope, especially the duodenum and the proximal small intestine. Endoscopic treatment was performed using four methods: the unroofing technique, endoscopic mucosal resection (EMR), EMR after precutting (EMR-P), and endoscopic submucosal dissection (ESD) [7]. In case of large and sticky lesions, endoscopic excision may technically be difficult and potentially increase the risk of bleeding and perforation [8]. Surgical resection is commonly recommended to remove the lead point, such as lipoma larger than 2 cm in diameter [9]. In such cases, surgical resection may be the preferred approach. Operative management is mainly divided into four procedures, local excision of the lipoma, single segment bowel resection, multiple partial bowel resection, and extensive bowel resection. The type of procedure performed depends on the patient’s condition as well as the tumour size, number and location.

Extensive resection of all multiple lipomas can cause short bowel syndrome. Therefore, determining the exact range remains a problem. However, with excision, the total tumour resection can be performed without resecting the intestinal tract regardless of the number of tumours. All lipomas were resectable by combining ESD and local excision with plastic repair as in this case. Although the procedure requires long duration and is complicated, it seemed to be one of the methods of resecting multiple lipomas because it prevents short bowel syndrome and allows total resection.

Lipomatosis is a benign disease, and mobility is good if it occurs in the small intestine; therefore, surgery under small laparotomy is indicated. Beside, in cases of multiple and diffuse lipomas, the entire position of the tumour cannot be determined preoperatively, and small umbilicus laparotomy would be an option in this case, considering resection, reconstruction, and suturing. However, as the duodenum has poor mobility and many adjacent organs were found, its surgical procedure becomes very complicated in cases of duodenal lipoma. Laparoscopic and robotic surgery may be suitable for duodenal lipoma, which is difficult to endoscopically resect. Laparoscopic approach is minimally invasive surgical procedure with minimal postoperative pain and short hospitalization [10]. Robotic technology has been shown to facilitate complex resection and reconstruction [11].

In the future, cases and indications of surgery and resection method for GI lipoma should be accumulated and considered, respectively.　

## 4 Conclusions

This is an unusual case of ileo-ileo intussusception caused by GI lipomatosis in an adult. Multiple local excision ± ESD seemed to be one of the methods in resecting multiple GI lipomatosis.More case reports and surgical approaches can establish future progress in surgery.

## Conflicts of interest

This case report did not receive any conflicts of interest.

## Funding

This case report did not receive any specific grant from funding agencies in the public, commercial, or not-for-profit sectors.

## Ethical approval

This case report was performed in compliance with the principles of good clinical practice outlined in the Declaration of Helsinki and federal guidelines. This case report was approved by the local ethic committee.

## Consent

This report was written informed consent was obtained from every participant or the participant’s guardian.

## Author’s contribution

Yasunori Yoshimoto, Takahisa Fujikawa, and Tsunenori Yamamoto greatly contributed to preoperative diagnosis, determination of surgical technique and surgery. Tomoharu Yoshida and Yasuyuki Shirai contributed to preoperative diagnosis and performed an endoscopic approach. Yasunori Yoshimoto, Tomoharu Yoshida were a major contributor in writing the manuscript and helped with the critical revision. All authors read and approved the final manuscript.

## Registration of research studies

This is a case report, not research studies.

## Guarantor

Yasunori Yoshimoto is the Guarantor.

## Provenance and peer review

Not commissioned, externally peer-reviewed
